# Live microscopy: cracking the challenge to image biology unfolding in cells, tissues, and organs

**DOI:** 10.1038/s42003-022-03601-8

**Published:** 2022-07-07

**Authors:** Marco Fritzsche

**Affiliations:** 1 Rosalind Franklin Institute, Harwell Campus, Didcot, OX11 0FA UK; 2 Kennedy Institute of Rheumatology, University of Oxford, Roosevelt Drive, Oxford, OX3 7FY UK

**Keywords:** Microscopy

## Abstract

*Communications Biology* is inviting submissions on the topic of live microscopy – from new tools to emerging techniques, from conventional to advanced light microscopy - with the aim of publishing high-quality research devoted to advance our understanding of biology.

Visualising biology through a microscope has been a powerful strategy for scientific discovery for many years. However, only the latest revolutionary technologies in advanced live cell microscopy demonstrate the technological leap long hoped for to overcome the challenges to image the inner workings of living cells, tissues, and organs free of any influence from the observer.

Over the last couple of years, we have all become more aware of the spatiotemporal structures and dependencies of biology. Without knowledge of the different cell types, their molecular characteristics, and their location in the body, we cannot understand the relationships of the biological and biophysical networks that underpin the physiology and behaviour of life. Aiming to map every cell type in the human body, the Human Cell Atlas is expected to transform our understanding of human physiology, and lead to major advances in the diagnosis, maintenance, and treatment of health and disease. This has been a major driver of investment in cutting-edge high-dimensional flow cytometry-based phenotyping, single-cell, spatial genomics, and computational bioinformatics technologies. This provides an unprecedented resource and benefits research communities and, ultimately, patients.

Around the world, researchers work hard to further develop bioimaging technologies^1–3^ and analysis techniques to correlate and interpret the large information volumes uncovered by these disparate data sources^4^. At the outset, this upwards trend and increasing importance of bioimaging and analysis technologies in the biomedical sciences, has more recently underlined the pressing demand for another revolutionary technological leap needed and capable to overcome the challenge to image the inner workings of life. Many studies have demonstrated that the complex relationships of biological systems, with their different cell types, diverse genetics, and the continuously evolving spatiotemporal tissue context, are of dynamic nature^5^. Living cells themselves are constantly measuring and interpreting changes in the biochemical and biophysical properties of their surroundings, and actively adapt to those.

These recent advances in biological research have highlighted not only the need of progressive live cell microscopy with the right sensitivity but, in particular, the importance of minimal invasiveness to the biology of interest. Bioimaging must be free of influence of the observer, particularly the instrument’s illumination, which has historically been challenging to achieve technically, but has significantly improved by the latest advanced live microscopes. The ambition to progress single-cell and tissue genomics to live measurements needs to resolve the paradox that the majority of established methodologies work best in fixed conditions.

The most promising advanced live cell microscopy technologies, including lightsheet, structured illumination, airyscan, and multi-photon techniques, are expected to shape the future of microscopy. Ultimately, with the vision to develop the ideal live cell microscopy platform with the highest technically-possible spatial and temporal sensitivity, and minimal invasiveness, the integration of these bioimaging technologies in a single biophotonic platform promises a future-proof strategy. One of the big tasks today is democratising and translating bespoke state-of-the-art methodologies to commercial platforms and thus making them available to all researchers in the biomedical and physical communities. Opportunities for collaboration with the microscopy industry, simply due to the magnitude of the challenge, is therefore imminent. Among many advantages and possibilities, a strategic partnership with leading microscopy companies that are committed to the science of microscopy, seems very attractive to accelerate the availability of leap microscopy technology. Similarly, fast progress in the corresponding software analysis tools require industry to step up as very little systematic investment has been seen in academia yet. The Advanced Imaging Center at the Howard Hughes Medical Institute Janelia Research Campus has been and continues to play a crucial role in accelerating availability by giving scientists access to cutting-edge bespoke new advanced microscopes and facilitate their broad availability through commercialization. A forward-looking view is that a modern state-of-the-art bio-imaging centre of excellence should offer both state-of-the-art commercial microscopy platforms and active development of new bespoke technology to support the best discovery science.

Consequently, current research must continue to advance live cell microscopy, both literally in the context of improving the spatiotemporal sensitivity and invasiveness to map biological processes across scales, and more figuratively in terms of involving research efforts from different perspectives. Across the fields of advanced microscopy hardware and software development as well as the biological sciences, there’s a strong commitment to crack the challenge of imaging biology unfolding in cells, tissues, and organs. We at *Communications Biology* are thus welcoming research article submissions that champion efforts in live cell microscopy and bioimaging analysis. Since our launch in January 2018, we have continuously been a venue for basic research that shapes the emerging interface where biology meets microscopy. With our call for submissions to this Collection, we aim to illustrate how the latest technologies in live cell microscopy demonstrate the long hope for technological leap that is capable of overcoming the challenge to image the inner workings of living cells, tissues, and organs free of any influence.

We are inviting submissions on (advanced) live cell microscopy with the intent of enhancing the visibility of current research investigating the ways in which light microscopy transforms biological research. In particular, we are interested in (but by no means limited to) studies of the light-sheet, structural illumination, airyscan, and multi-photon microscopy. We are also keen to receive studies focusing on tool and probe development, as well as label-free microscopy. Recent examples are the study by Chen et al.^6^, exemplifying lightsheet and three-dimensional super-resolution imaging^6^. Our Collection will be handled by our Editorial Board members who are experts in imaging techniques, tools and microscopy development, such as Natalie Elia (Ben Gurion University, Israel), Periklis Pantazis (Imperial College London, UK), Chao Zhou (Washington University in St.Louis, USA) and myself.

As we at *Communications Biology* set out to grow and develop this Collection, we would like to think of you as a crucial contributor to making this a success story. We hope that our Collection will contribute to the future direction the live cell microscopy community is taking.
