# Author Correction: Live microscopy: cracking the challenge to image biology unfolding in cells, tissues, and organs

**DOI:** 10.1038/s42003-022-03687-0

**Published:** 2022-07-18

**Authors:** Marco Fritzsche

**Affiliations:** 1 Rosalind Franklin Institute, Harwell Campus, Didcot, OX11 0FA UK; 2 Kennedy Institute of Rheumatology, University of Oxford, Roosevelt Drive, Oxford, OX3 7FY UK

**Keywords:** Microscopy

Correction to: *Communications Biology* 10.1038/s42003-022-03601-8, published online 07 July 2022.

In the original version of the Article, the fourth paragraph incorrectly stated “These recent advances in biological research have highlighted not only the need of progressive live cell microscopy with the right sensitivity but, the importance of minimal invasiveness to the biology of interest.” The text should read, “These recent advances in biological research have highlighted not only the need of progressive live cell microscopy with the right sensitivity but, in particular, the importance of minimal invasiveness to the biology of interest.”

In addition, the fifth paragraph incorrectly stated “he most promising advanced live cell microscopy technologies, including lightsheet, structured illumination, airyscan, and multiphoton techniques, are expected to shape the future of microscopy.” The text should read, “The most promising advanced live cell microscopy technologies, including lightsheet, structured illumination, airyscan, and multiphoton techniques, are expected to shape the future of microscopy.”

Lastly, the sixth paragraph incorrectly stated “We at *Communications Biology are thus welcoming* research article submissions that champion efforts in live cell microscopy and bioimaging analysis.” The text should read, “We at *Communications Biology* are thus welcoming research article submissions that champion efforts in live cell microscopy and bioimaging analysis.”

This has now been corrected in the PDF and HTML versions of the Article.

